# More Prosocial, More Ephemeral? The Role of Work-Related Wellbeing and Gender in Incubating Social Entrepreneurs’ Exit Intention

**DOI:** 10.3390/ijerph19073999

**Published:** 2022-03-28

**Authors:** Jianing Dong, Xiao Wang, Xuanwei Cao, David Higgins

**Affiliations:** 1 International Business School Suzhou, Xi’an Jiaotong-Liverpool University, Suzhou 215123, China; jianing.dong20@student.xjtlu.edu.cn (J.D.); xuanwei.cao@xjtlu.edu.cn (X.C.); 2 Management School, University of Liverpool, Liverpool L69 3BX, UK; dhiggins@liverpool.ac.uk

**Keywords:** social entrepreneur, entrepreneurial exit intention, prosocial motivation, wellbeing, gender

## Abstract

Why does social entrepreneurship tend to live so shortly? A range of studies tried to answer this question, although very few delved into the “inner layer” (psychological status) to unveil how social entrepreneurs decide to quit. Accordingly, focusing on prosocial motivation of social entrepreneurs and its impact on their work-related wellbeing and then their business exit intention, we conducted this empirical research. Furthermore, gender differences are involved based on relevant calls for in-depth investigation. With a sample of 301 respondents in China, deploying the partial least square structural equation modeling (PLS-SEM), we found prosocial motivation decreases entrepreneurs’ work-related wellbeing, which in turn, increases entrepreneurial exit intention. Furthermore, adopting the multi-group analysis (MGA) technique, we uncovered that the impact of prosocial motivation on work-related wellbeing largely is stronger for males. Our research thus contributes to the growing research and knowledge on social entrepreneurship in terms of individual personality traits and how they impact a social entrepreneur’s psychological status and thus their intention of exiting the social business. This study’s further theoretical and practical implications, as well as its limitations and thus future research directions, are discussed at the end.

## 1. Introduction

While most entrepreneurial studies focus on successful entrepreneurs, quite a few shed light on unsuccessful ones who quit their businesses [1,2], especially social entrepreneurs who endure more difficulties in sustaining their businesses [3,4]. Despite the fact that anyone who starts a business will, in the course of time, exit his or her firm [1,5,6], little is known about the psychological antecedents of such a personal decision of exit [1,7,8,9] and, more importantly, the emotional processes that might be undermining the business operation, eventually leading to the exit decision [8,10].

One of the psychological antecedents that attracts great research interest in the entrepreneurial exit decision is prosocial motivation [11]. Prosocial motivation is “the desire to benefit others or expend effort out of concern for others” [12]. Social entrepreneurs refers to the entrepreneurs who are aware of the importance of social value creation and who embed relevant activities into their business activities to highlight the importance of creating social value with their business endeavors, thus providing positive externalities to society at large [13,14,15,16,17]. The main distinctive quality of social entrepreneurs—their prosocial motivation—is what differentiates them from conventional or regular entrepreneurs [18]. However, to our surprise, the research findings, on the relationship between prosocial motivation and entrepreneurial exit in the context of social venturing, are controversial.

Social entrepreneurs who embed prosocial motivation into their entrepreneurship may encounter various challenges and may be confronted with significant obstacles or resistance in persisting their business than regular or commercial entrepreneurs [3,19]. For example, as “social goals distract from the pursuit of economic viability” [4,20], social entrepreneurs usually struggle to obtain sufficient funding [21,22], and are less likely to be successful in the development of a viable firm than the entrepreneurs whose main work motivation is based on financial goals [3], leading to the entrepreneurial exit [23]. However, Grant [24] suggested that when an individual such as an entrepreneur with prosocial motivation has direct contact with the beneficiaries of his or her work, the experiences become emotionally charged, and he or she is more affectively engaged in the work and likely to sustain their career. Mcmullen and Bergman [25] also indicated a similar view in a case study that prosocial entrepreneurs are like the members of an organization, producing “attachment to the organization” in the process of helping others through work, which in turn “makes their exit emotionally difficult”. These conflicting opinions imply that an understanding of the relationship between prosocial motivation and entrepreneurial exit is still deficient.

Maybe it is challenging to attribute entrepreneurial exit to prosocial motivation alone, since prosocial motivation is a personality trait [26] that unnecessarily triggers actual behavior; therefore, its capacity to predict the exit can be quite limited [27,28,29,30,31]. Likewise, the hierarchical approach to personality has questioned the direct relationship between personality traits and behavioral outcomes [31,32,33]. In fact, Rauch and his colleagues argued that personality traits are associated with behavioral outcomes through more specific mediating processes [28]. Additionally, Lindblom, Lindblom, and Wechtler [31] suggested bringing in a mediator that can play an essential role in the relation between prosocial motivation and exit intention.

Prior exploration has suggested that work-related wellbeing could act as this kind of mediator between relatively abstract personality traits and comparatively tangible work-related outcomes [14]. On the one hand, Judge and Larsen [34] found that the nature and structure of job attitudes (in this research, work-related wellbeing) “have a strong affective and dispositional basis”. Extensive theoretical and empirical research substantiates the view that a significant part of job attitudes is rooted in personality traits [34,35]. On the other hand, Steel [36] found that “no other single domain of work has had as much influence on turnover research as attitude theory does”. Boswell et al. [37] claimed that work attitudes consistently emerge as important factors in predicting exit. Based on the views mentioned above, this study proposes that studying work-related wellbeing as a potential mediator could provide a new significant understanding of the effects of prosocial motivation on entrepreneurial exit. Mostly, work-related wellbeing is regarded as a three-dimensional umbrella term comprising job satisfaction, work anxiety, and work burnout [14,38,39]. These three dimensions of work-related wellbeing likely provide more details on how social entrepreneurs might develop positive or negative feelings about their work, due to the prosocial motivation, and thus affect their entrepreneurial exit.

Meanwhile, a special GEM (global entrepreneurship monitor) report dedicated to social entrepreneurship [40] claims that social ventures/enterprises, entailing the integration of social welfare and commercial aims in an organization’s core values [41], are more likely to be started up by males than females, whereas the gender gap in the later stages of the entrepreneurial life cycle is not as broad. Prior studies suggested that males and females perceive the social businesses around them with “different eyes” [42,43]—male entrepreneurs give up personal identification pertaining to social entrepreneurship more easily than female entrepreneurs [43]. This type of difference in cognitive processes and thus perceptions influences their decisions on starting up a business with social value creation, leading to the gender gap in social entrepreneurial activities [44]. Nonetheless, it still cannot sufficiently explain why female entrepreneurs persist in social entrepreneurship more than male entrepreneurs [14,45].

This study, therefore, is to respond to prior studies’ calls for investigating why current research characterizes social entrepreneurs with heroic attributes [46] rather than recognizing that they are enduring more difficulties in sustaining their social enterprises [3,4]. In spite of few studies exploring the relationship between prosocial motivation of social entrepreneurs and their exit intention [3,24,25], the conflicting arguments imply substantial gaps and debates in our understanding. Inspired by Rauch et al.’s suggestion of probing more specific mediating processes between prosocial motivation and exit intention [28], this study involves work-related wellbeing (three dimensions: job satisfaction, work anxiety, and work burnout). Meanwhile, given the significant gender gap in social entrepreneurial activities as well as its latent influences on the effect of prosocial motivation on work-related wellbeing, this study also examined such moderating effects.

With the cross-sectional survey data of 301 social entrepreneurs in China, and PLS-SEM technique suggested by Ringle et al. [47] and run by SmartPLS (v.3.3.3), this research examined how their prosocial motivation affects exit intention, mediated by work-related wellbeing (three dimensions: job satisfaction, work anxiety, and work burnout). This research finds that prosocial motivation’s effect on exit intention is separately mediated by job satisfaction and work anxiety; furthermore, prosocial motivation’s effect on job satisfaction and work anxiety are both stronger for male social entrepreneurs than female.

These findings may have several contributions to the existing literature. First, our study particularly extends the theoretical work of Tina, Foss, and Stefan [11], which called for exploring whether entrepreneurs’ motivation to do good for others incurs negative consequences for themselves, especially entrepreneurial exit. In doing so, this research echoes an emerging critique that so far much of the social entrepreneurship literature focuses on individual social entrepreneurs and tends to characterize these individuals with heroic attributes [46]. In view of Light [48], this heroic characterization and focus on the stories of individual success largely limits our capacity to learn from the processes of entrepreneurial failure. Second, this study extends recent research on the relationship between social entrepreneurs’ prosocial motivation and exit intention [3,24,25] by involving the role of entrepreneurs’ work-related wellbeing, and by developing our understanding of what types of work-related wellbeing influence their exit intention. Third, this research discusses new avenues of including gender role theory and gender stereotypes for further probing the relationship between prosocial motivation and work-related wellbeing as well as its effect on exit intention of social entrepreneurs.

The next section elaborates on the theoretical background and hypothesis development, followed by the data collection and methodology. Presenting the results, this paper discusses the theoretical and practical implications, limitations, and future research directions, followed by the conclusion.

## 2. Theoretical Background and Hypothesis Development

Entrepreneurial exit is an outcome and integral part of the entrepreneurial process [49]. Lindblom, Lindblom, and Wechtler [31] and Pollack, Vanepps, and Hayes [49] defined entrepreneurial exit intention as “an entrepreneur’s desire or goal, at some point in the future, to leave his or her venture”, which largely is based on assessing the willingness to continue his or her work as an entrepreneur. Even though it is not always explicitly claimed, one research direction that tends to explicate the antecedents of entrepreneurial behaviors is theoretically grounded in the school of personality traits [50] (see Ajzen, 1987 for a review). Furthermore, as the predisposition for acting [11,50,51], personality traits largely affect how people remember, perceive, and interpret events, and thus how they ultimately behave [52,53].

In this study, prosocial motivation, as a stable personality trait and the main distinctive quality of social entrepreneurs [18], represents “a person’s ‘affective lens’ (remains constant over the time) on the world” [12,26,35], determining their responses in various contexts and shaping their behaviors [32,33]. Prosocial motivation determines the value, purpose, and meaning as motives of effort from an altruistic and humanitarian perspective [54]. Without prosocial motivation, few (if any) people would pay attention to social entrepreneurship, and most social problems would remain unsolved due to a lack of economic incentive [25,55,56]. However, prosocial motivation may incur extra burdens or pressures that eventually lead to escalating intention of exit [12,20,57].

### 2.1. Prosocial Motivation and Exit Intention

In most of the relevant studies, findings suggested that entrepreneurs with strong prosocial motivation may decrease their intention of exiting from their social ventures. Some of them revealed a direct effect [57], while others uncovered a buffering effect such that prosocial motivation discourages entrepreneurial exit intention by creating “attachment to organization” [24,25].

Compared with the brightness of prosocial motivation, very few have investigated its dark side [12]. To date, most studies claim that social entrepreneurs are heroic, but the “heroes” also encounter a range of adversities in running businesses [58,59,60,61], and mostly heroic stories end tragically. According to prior research, entrepreneurs with strong prosocial motivation are less likely to be successful in the development of a viable firm than the ones whose main work motivation is based on financial goals [3]. This kind of failure is equivalent to entrepreneurial exit [23], but few have investigated the mechanism. Thus, addressing the debate of prosocial motivation’s effect on entrepreneurial exit and echoing the calls for developing a broader understanding of the “dark side” of prosocial motivation [12], this research investigates the relationship between prosocial motivation and social entrepreneurs’ intention of exit as well as the potential mechanism.

According to the school of personality traits, individuals’ prosocial motivation is a stable personality trait, participating in emotional reactions and influencing ultimate behaviors [11,50,51]. Entrepreneurial exit is an emotional process that may involve the experience of negative emotions [62,63]. Thus, without unveiling such an impact of prosocial motivation on emotional status, studying how prosocial motivation can influence social entrepreneurs’ intention of exit could be fruitless. In fact, Rauch and Frese [28] highlighted the “left room” for mediating variables between prosocial motivation and entrepreneurial exit intention, implying that prosocial motivation locates relatively far from entrepreneurial exit intention, and researchers should consider indirect effects between prosocial motivation and exit intention [28]. Based on the aforementioned findings on the emotional factors of exit intention [31,64,65,66], this research argues that by affecting job satisfaction, work anxiety, and work burnout (the three dimensions of work-related wellbeing), prosocial motivation fosters the entrepreneurial exit intention.

### 2.2. Work-Related Wellbeing

Research on the wellbeing of entrepreneurs who seek to create social value is rare [67], especially when their work is pertinent to social value creation [14]. Work-related wellbeing is an umbrella term to explain an attitude (positive or negative) towards ranking their job or job situation [68,69,70,71]. Work-related wellbeing was conceptualized as a pervasive and persistent attitude and affective and cognitive state in three dimensions: job satisfaction, work burnout, and work anxiety [14,37,38,39,72,73,74,75,76]. The three dimensions seem interrelated, but they can be independent of each other [77]. For example, a person may experience their work as difficult and demanding (low job satisfaction) and may suffer from performance anxiety (high anxiety), but still feel enthusiastic (low burnout) about work [78]. Although social entrepreneurs may have a higher level of prosocial motivation, with a low level of work-related wellbeing, inevitably they may consider terminating their work for restoring the lost wellbeing, thus leading to escalation of the exit intention.

### 2.3. Prosocial Motivation, Job Satisfaction, and Exit Intention

Job satisfaction is commonly defined as an attitudinal evaluative judgment of one’s job or job experiences [79]. Prior studies have uncovered a positive relationship between prosocial motivation and job satisfaction [80,81]. Notwithstanding, as implied by Bolino and Grant [12] and Shepherd [82], entrepreneurs’ motivation to do good for others may generate negative effects on entrepreneurs themselves. Based on Bolino and Turnley [83], from the perspective of psychological costs of prosocial motivation, Grant [54] found that when individuals expend additional effort at work to fulfill their motivation to help others, they can experience work overload and increased levels of stress, which is also suggested by Vansteenkiste et al. [84]. Since prosocial motivation is a stable personality trait [11,50,51], the prosocial individuals (e.g., social entrepreneurs) tend to suffer high-level pressures frequently [85], which in turn can decrease their job satisfaction [86,87].

As high-level job satisfaction implies high-level productivity and low-level absence [1,88,89], job satisfaction tends to have a considerable negative effect on the exit intention [90,91]. By comparison, low-level job satisfaction likely leads to low-level productivity and high-level absence, thus discontinuing the current job will be inevitable. Based on the arguments above, prosocial motivation can incur extra burdens and pertinent pressures [92,93], decreasing job satisfaction. The deteriorated job satisfaction can undermine the job productivity and efficacy, leading to latent escape, job absence, and intention of exit. Therefore, this research hypothesizes:

**Hypothesis** **1a.** *Prosocial motivation is negatively related to job satisfaction.*

**Hypothesis** **1b.** *Job satisfaction mediates the relationship between prosocial motivation and entrepreneurial exit intention.*

### 2.4. Prosocial Motivation, Work Anxiety, and Exit Intention

Work anxiety is defined as an emotional state of perceived apprehension and increased arousal [94,95]. Previous research suggested that entrepreneurs with strong prosocial motivation are less likely to be successful in the development of a viable firm than entrepreneurs whose main work motivation is based on financial goals [3], since entrepreneurs with prosocial motivation are considered to be confronted with distinct challenges when establishing their ventures. For example, regarding entrepreneurs with a high level of prosocial motivation, considerable research has highlighted the challenges in attracting financial capital [58,59,60,61]. A large-scale survey in the UK indicated that attracting financial capital is perceived as a considerable challenge to venture growth among social entrepreneurs [96]. Largely, adequate income, especially the availability of financial resources, is a buffer against the anxiety and psychological strain of running the business, and vice versa [97,98,99,100]. Studies have proved that entrepreneurs who suffer from persistent financial difficulties, especially when financial difficulties become an obvious signal of entrepreneurial failure [4], will find their job responsibilities arduous or overwhelming [101] and feel they have little control over many things [102], thus leading to work anxiety [4,96,103].

Meanwhile, empirical research commonly supports the relationship between work anxiety and exit intention for prosocial careers. For instance, academics have indicated that work anxiety predicts exit intention amongst some occupations with prosocial characteristics, such as professionals of emergency medical services (EMS) [104,105], governmental employees [106], and teachers [107], especially when their prosocial goals lack a sense of being respected (e.g., insufficient workforce, low wages, and long working hours). For the entrepreneurs who embed social value creation into economic activities, the tension between the two types of business goal largely incurs conflict [108]. When the social value creation cannot be appreciated as expected, their work anxiety can be increased, and thus the intention to quit can be strengthened for restoring the weakened wellbeing. Based on the arguments above, more prosocial motivation can cause more work anxiety due to more challenges, leading to a greater intention of work cessation for wellbeing restoration. Therefore, this research hypothesizes:

**Hypothesis** **2a.** *Prosocial motivation is positively related to work anxiety.*

**Hypothesis** **2b.** *Work anxiety mediates the relationship between prosocial motivation and entrepreneurial exit intention.*

### 2.5. Prosocial Motivation, Work Burnout, and Exit Intention

Work burnout refers to the condition of physical and emotional exhaustion, as well as the associated negative attitudes resulting from the intensive interaction of working with people [109]. Business owners with prosocial motivation are commonly outcome-focused; in other words, these business owners have a strong desire to achieve both their business’ profit goals and their individual social goals [110,111]. The willingness to concurrently pursue both goals can drive the business owners to engage in too many activities [54], therefore leading to a range of difficulties, such as the indispensable attention to the dual goals, which in turn can undermine the expected dual performance [112,113,114]. This not only impairs the potential capacity to expend resources to pursue both economic and prosocial targets [12,82], but also overstretches the personal resources needed and thus creates a burnout experience [20].

Meanwhile, a large number of studies reveal that work burnout has a positive effect on exit intention, by creating emotional exhaustion and overextension at work as well as increasing feelings of frustration and tension about future work performance [115,116,117]. Furthermore, this emotional exhaustion might be exacerbated when the social goals distract most resources and attention from the pursuit of economic viability [22]. Based on the arguments above, prosocial motivation can incur work burnout resulting from limited resources and attention, and the work burnout can create emotional exhaustion, leading to the intention of exit. Therefore, this research hypothesizes:

**Hypothesis** **3a.** *Prosocial motivation is positively related to work burnout.*

**Hypothesis** **3b.** *Work burnout mediates the relationship between prosocial motivation and entrepreneurial exit intention.*

### 2.6. The Moderating Role of an Entrepreneur’s Gender

Gender role theory [118] suggests that individuals have common expectations regarding the appropriate conduct for men and women. Therefore, individuals who do not conform to the “common expectation” will be challenged by societal norms regarding gender role stereotypes. Gender role stereotypes predominantly categorize work into feminine and masculine [119,120]. As a consequence, individuals tend to pursue the work that fits their socially recognized gender and evade the work viewed as suited to the opposite gender [119,120]. Gender stereotypes potentially regulate how males and females should behave [120].

Prosocial behavior is barely related to bold, risk-taking, and aggressive behavior; rather, it is related to empathy and a sense of social responsibility [51,121]. However, those prosocial behaviors and values are commonly more associated with females than males [122,123]; namely, females better fit the social entrepreneurs’ gender role stereotype than males. If an individual has a social identity that is targeted by a negative stereotype in a given situation with implications of reducing wellbeing and one’s sense of belonging, a stereotype threat arises [120]. Accordingly, if a male entrepreneur tries to create more social value with his business than commercial value, he likely feels marginalized and distressed in terms of contradicting his gender role stereotype, since females better fit the social entrepreneur gender role stereotype than males [43]. These concerns are essential to the psychological experience of stereotype threat, leading to declination of job satisfaction [124] and escalation of burnout [125] and anxiety [126] in stereotype-relevant contexts [127].

Accordingly, for male social entrepreneurs, the negative relationship between prosocial motivation and job satisfaction may be strengthened, as their additional effort to help others may conflict with their gender stereotype that is based on power, social status, and wealth. This, in turn, can increase the job tension incurred by the prosocial motivation [20], amplifying the negative effect of prosocial motivation on job satisfaction. Comparatively, for female entrepreneurs, the negative relationship between prosocial motivation and job satisfaction may be weakened, as their additional effort to help others likely fits their gender stereotype that is related to empathy, care, concern, and love. This, in turn, can decrease the job tension incurred by the prosocial motivation [43,128], ameliorating the negative effect of prosocial motivation on job satisfaction.

Similarly, for male social entrepreneurs, the positive relationship between prosocial motivation and work anxiety may be strengthened, as earning less than a regular (non-social) entrepreneur may conflict with their gender stereotype as a “breadwinner” [129]. This, in turn, can decrease the income security incurred by engaging in social entrepreneurship [130], amplifying the positive effect of prosocial motivation on work anxiety. Comparatively, for female entrepreneurs, as the gender stereotypes regard females as “care givers”, the lower income security incurred by engaging in social entrepreneurship would not substantially amplify the positive effect of prosocial motivation on work anxiety.

Due to social entrepreneurs’ difficulties in balancing resources for both economic and social goals [3], the regulation by gender stereotypes for males may amplify the effect of prosocial motivation on work burnout by escalating the conflicts of achieving both profit goals and social goals [125]. Comparatively, for female entrepreneurs, the positive relationship between prosocial motivation and work burnout may be weakened, as their gender stereotype determines that female entrepreneurs mostly are less likely to be successful in the development of a viable firm [131,132]. This, in turn, can somewhat mitigate the goal conflicts incurred by the prosocial motivation [12,67,133], alleviating the effect of prosocial motivation on work burnout. Based on the arguments above, this research hypothesizes:

**Hypothesis** **4a.** *The relationship between prosocial motivation and job satisfaction is stronger for male entrepreneurs.*

**Hypothesis** **4b.** *The relationship between prosocial motivation and work anxiety is stronger for male entrepreneurs.*

**Hypothesis** **4c.** *The relationship between prosocial motivation and work burnout is stronger for male entrepreneurs.*

**Figure 1:**
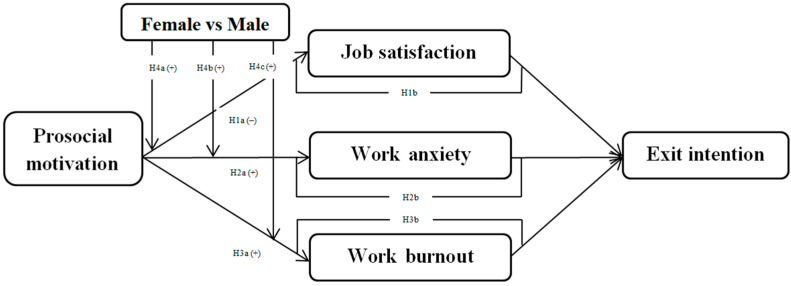
Theoretical model.

Figure 1 shows the theoretical model.

## 3. Method

### 3.1. Sample and Procedure

To test the hypotheses, we collected the primary data through an online survey of Chinese entrepreneurs in 2021, with the help of All-China Federation of Industry and Commerce (ACFIC). This organization is a semi-official organization of private firms that consists of business owners from various industries and firms of different sizes across China, and operates at the national, provincial, municipal, and county level. This study collected the data during the two large-scale colloquiums for entrepreneurs organized by ACFIC from July (location: Jinan, Shandong province, China) to August (location: Qingdao, Shandong province, China) in 2021. The invitation (on paper), including a QR code linking to an online questionnaire, was sent to entrepreneurs in the colloquiums.

Since the questionnaire from prior research was originally developed in English, this research adopted the approach suggested by Brislin [134] for the translation. After the questionnaire draft was completed, a pilot test was performed (*n* = 50) to check whether it was necessary to make any adjustment. Finally, with complementary literature review and interviews, 22 items for five constructs were eventually selected. The Cronbach’s alpha values of the pre-test were over 0.7 (Prosocial Motivation = 0.850; Work Anxiety = 0.944; Job Satisfaction = 1.000; Work Burnout = 0.962; Exit Intention = 0.946). According to the criteria of Nunnally [135], the internal consistency and stability of the questionnaire used for this study were acceptable.

A total of 450 entrepreneurs took the survey via the online questionnaire, of which the overall response rate exceeded 80%. This is an acceptable rate and similar to previous research [136]. This research identified social entrepreneurs according to a binary variable which was adopted by the GEM report: “Are you, alone or with others, currently trying to start or currently owning and managing any kind of activity, organization or initiative that has a particularly social, environmental or community objective? This might include providing services or training to socially deprived or disabled persons, using profits for socially-oriented purposes, organizing self-help groups for community action, etc.” Entrepreneurs marking “no” were identified as commercial entrepreneurs and excluded from this research; entrepreneurs choosing “yes” were recognized as social entrepreneurs and included in this research [137]. This method is adopted by other research into social entrepreneurship [138,139].

Unusable samples with many missing or apparently problematic values were removed to improve the statistical quality. Eventually, the sample size of this research was 301. Female participants accounted for 51.8% while male for 48.2%. Table 1 shows an overview of the sample demographics.

### 3.2. Variables and Measurement

Dependent variable: Exit intention. This research measured entrepreneurs’ exit intention using the three items developed by Pollack, Vanepps, and Hayes [49], and the participants responded to each of the items on a Likert 7-point scale ranging from 1 (strongly disagree) to 7 (strongly agree).

Independent variable: Prosocial motivation. This research measured the extent to which entrepreneurs have prosocial motivation with the four items adopted by Grant [54], and the participants responded to these items on a Likert 7-point scale ranging from 1 (strongly disagree) to 7 (strongly agree).

Mediating variable: Job satisfaction. This research measured entrepreneurs’ job satisfaction with a single item based on a Likert 7-point scale ranging from 1 (strongly disagree) to 7 (strongly agree), developed by Chordiya et al. [140]: “Generally speaking, I am satisfied with my job”. Extensive studies measured job satisfaction with this single-item measurement [140,141,142] and recognized its advantages [143,144].

Mediating variable: Work anxiety. This research measured work anxiety using the four-item scale developed by Haider et al. [145]. Entrepreneurs responded to each of the items on a Likert 7-point scale ranging from 1 (strongly disagree) to 7 (strongly agree).

Mediating variable: Work burnout. This research measured work burnout using the ten-item scale developed by Malach-Pines and Ayala [146]. Entrepreneurs responded to each of the items on a Likert 7-point scale ranging from 1 (strongly disagree) to 7 (strongly agree).

Moderating variable: Gender. Male respondents were coded as “1” and female respondents were coded as “2”.

The English questionnaire has been appended, presenting details of all the measurements (Appendix A).

### 3.3. Measurement of Control Variables

Following most entrepreneurship studies, this research included several demographic variables as the control variables (detailed in Table 1) such as age [131] and educational achievement (1 = junior high school, 2 = high school or equivalent, 3 = junior college, 4 = bachelor degree, and 5 = postgraduate or above) [147]. Among the control variables, involving age and education was due to Mayr and Freund [148] and Harding [149] suggesting their significant impact on sustaining social entrepreneurship.

## 4. Data Analysis

Manley et al. [150] pointed out that partial least squares structural equation modeling (PLS-SEM) is increasingly important for entrepreneurship research. PLS is appropriate for uncovering causal relationships among constructs and can deal with the model constructs and measurement items synchronously, less limited to the randomness and normality of variables in research. Thus it can facilitate complex models [150,151,152] unveiling the relationships between variables with abnormal data distribution.

Specifically, there are three reasons for adopting PLS-SEM for this research: first, PLS-SEM is a prediction-oriented method suitable for studying what has not been well tested before [153]—in this case, lack of knowledge or studies about the relationship between prosocial motivation and entrepreneurial exit intention. Second, PLS-SEM has been proved to be effectual for empowering and testing complicated models [152]. As this research examines the complex relationship between prosocial motivation, job satisfaction, work burnout, work anxiety, and exit intention, and in order to decrease measurement error and avoid collinearity, PLS becomes more suitable for this research than other SEM methods. Third, in this research, the samples size is 301, meeting the criterion of Majchrzak et al. [154] that the sample size should be at least 5 to 10 times the maximum number of model paths (in this research the maximum number of model paths is 3).

In terms of the common method variance (CMV) issue, this research deployed Harman’s one-factor test for the CMV issue [155]. The result of exploratory factor analysis for the first factor (35.44%) indicates that CMV is not an issue in this study.

Deploying PLS-SEM, this research followed the two-step approach [156]: the first step is to assess the outer model and the second step is to examine the inner model that tests the measurement model and structural model, respectively.

### 4.1. Outer Model and Scale Validation

The basic evaluation of the outer model included the reliability of each item and the internal consistency, convergent validity, and discriminant validity of each construct.

The reliability of each item was tested by the factor loading that the recommended threshold value is 0.5 [157]. The results of factor loading are presented in Table 2 and all the results are above the recommended threshold value.

The results of the internal consistency test are indicated by the composite reliability (CR) and Cronbach’s alpha. Table 2 shows all values of composite reliability are above the recommended threshold value of 0.8 [157] and Cronbach’s alpha values are above 0.60, showing that the best validity measurement explains the scale’s structure and the overall consistency level is high [135].

The convergent validity is indicated by the average variance extracted (AVE) for each construct, and the recommended threshold value is 0.5 [157]. It is seen from Table 2 that the AVEs for potential variables of the constructs in this study are between 0.657 and 1.000, indicating good convergent validity.

The discriminant validity was assessed by two criteria. First, the heterotrait–mono-trait ratio (HTMT) of correlations introduced by Henseler et al. [158], which is based on the multitrait–multimethod matrix used to test the discriminant validity. When the HTMT ratio is below 0.90, the discriminant validity can be accepted [159]. Table 3 shows that the HTMT ratios range between 0.053 and 0.575, indicating that discriminant validity has been established.

Second, as shown in Table 4, the comparison of cross-loadings and factor loadings for each indicator shows reasonable discriminant validity, when the factor loading of each scale item for its assigned latent construct is higher than its loading on any other constructs, also indicating the discriminant validity has been established [160].

### 4.2. Inner Model and Hypotheses Testing

**Figure 2:**
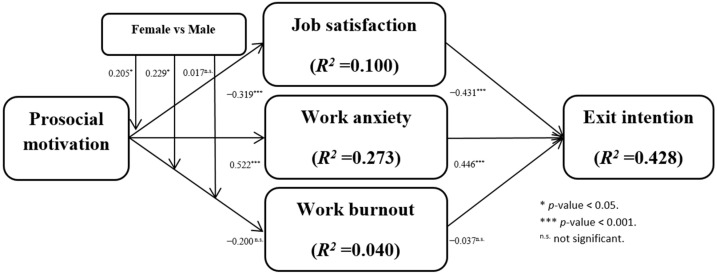
Path coefficients and R-squares of the inner model.

The inner model of PLS-SEM was involved to estimate the path coefficients, R^2^ and Q^2^. Path coefficients imply the magnitude and direction of the variable relations. According to Chin [151], this research used the bootstrapping method (5000 bootstrap samples) to evaluate the significance of each path coefficient. Based on the *p*-values in Figure 2 and Table 5, one path (H3a: PM→WB) was rejected and the other two paths were supported. Specifically, Figure 2 and Table 5 show that prosocial motivation negatively and significantly affect job satisfaction, supporting H1a (PM→ JS: β = −0.319, *t*-value = 6.330); prosocial motivation positively and significantly impacts work anxiety, supporting H2a (PM→WA: β = 0.522, *t*-value = 8.825); prosocial motivation does not have a significant impact on work burnout, indicating that H3a is not supported (PM→WB: β = −0.200, *t*-value = 1.318).

Figure 2 shows that the values of R^2^ for job satisfaction (R^2^ = 0.100), work anxiety (R^2^ = 0.273), work burnout (R^2^ = 0.040), and exit intention (R^2^ = 0.428) have explanatory power.

Meanwhile, in this research, the values of Q^2^ for job satisfaction (Q^2^ = 0.094), work anxiety (Q^2^ = 0.201), work burnout (Q^2^ = 0.006), and exit intention (Q^2^ = 0.357) are larger than zero, suggesting that the theoretical model of this study has sufficient explanatory power.

### 4.3. Testing of Mediation Effects

This research tested for the mediation of job satisfaction, work anxiety, and work burnout separately. The bias-corrected bootstrap with 95% confidence interval (CIs) based on 5000 bootstrap samples for the indirect effect indicates that job satisfaction and work anxiety separately mediate the relationship between prosocial motivation and exit intention. Therefore, hypothesis H1b and H2b are supported. The mediation of work burnout between prosocial motivation and exit intention is not statistically significant, resulting in the rejection of hypothesis H3b.

Hair Jr., Hult, Ringle, and Sarstedt [160] suggested that to determine the strength of the indirect effect (i.e., mediating effect) in relation to the total effect (i.e., direct effect + indirect effect), the variance accounted for (VAF) is informative. According to [160], if VAF > 80%, it is full mediation; if VAF ≤ 80%, it is partial mediation; if VAF < 20%, there is no mediation. Table 6 indicates that job satisfaction and work anxiety are significant partial mediators between prosocial motivation and exit intention (both the VAF values range from 42.9% to 50.8%), whereas work burnout is not a significant mediator (VAF < 20%). Therefore, H1b and H2b are supported and H3b is not supported. Additionally, compared with job satisfaction, work anxiety produces a greater mediating effect.

### 4.4. Multi-Group Analysis

The inner model was validated across the genders using multi-group permutation tests [161]. Table 7 displays the assessment results of the measurement model between the two datasets of male social entrepreneurs (n = 145) and female social entrepreneurs (n = 156). The path coefficients (β) have been estimated, and differences of the two coefficients have been analyzed and found to be significant (H4a: βdiff = 0.214, *p* = 0.046; H4b: βdiff = 0.229, *p* = 0.044). The results show that job satisfaction and work anxiety are more strongly affected by prosocial motivation for males than for females. Therefore, H4a and H4b are supported.

The path coefficient for H4c is not statistically significant, implying gender does not have a significant moderating effect on the relationship between prosocial motivation and work burnout [162]. Therefore, H4c is not supported.

## 5. Discussion

Responding to the call for in-depth research into unsuccessful entrepreneurs, especially social entrepreneurs who largely endure far more difficulties [58,59,60,61], we delved into the relevant literature and recognized the necessity and significance in exploring the antecedent and potential mechanism of entrepreneurial exit [1,163]. Specifically, suggested by the studies on exit intention [3,23,25] and those questioning the direct effect of prosocial motivation on exit intention [12,27,28,29,30,31] as well as those implying the necessity of investigating the role of gender, this research involved job-related wellbeing as a mediator and gender as a moderator, exploring how prosocial motivation respectively affects the three dimensions of job-related wellbeing, how they separately influence the exit intention, and what roles the gender stereotypes play.

### 5.1. Theoretical Implication

With a sample size of 301 observations and the consistent bootstrapped PLS-SEM estimations, this research finds that prosocial motivation has a negative relationship with job satisfaction and job satisfaction mediates the nexus between prosocial motivation and exit intention. This finding is remarkable as it challenges prior studies [80,81] that claim greater prosocial motivation is positively related to individuals’ job satisfaction if individuals perceive that they can contribute to society and do good for other people with their current jobs [80,81]. This difference is probably due to the sample composition. Most respondents in the prior studies’ samples were public professionals who had direct contact with the beneficiaries in their work, facilitating trust development between benefactors and beneficiaries. This can obscure the proposed negative relationship between prosocial motivation and job satisfaction [80]. In contrast, social entrepreneurs largely are far away from beneficiaries in their social work, as this kind of entrepreneur mostly helps beneficiaries indirectly through initiatives and organizations or managing activities, rather than directly contacting the beneficiaries [24,25]. This “distance”, therefore, can restrict social entrepreneurs achieving such job satisfaction. In addition, as the prior studies were conducted in the context of Western economies while this study is in China, the impact of prosocial motivation on job satisfaction may vary in the latter context due to its distinction and complexity, which in turn necessitates further studies.

This research also finds that prosocial motivation is positively related to work anxiety that mediates the link between prosocial motivation and exit intention, indicating that entrepreneurs with greater prosocial motivation are more anxious about their work, leading to escalation of the exit intention. This finding is remarkable since no prior studies have specifically investigated such a nexus between prosocial motivation and work anxiety. Such a relationship might be due to the fact that the costs of prosocial behaviors tend to emerge quickly, whereas the benefits mostly are uncertain and can be recognized after a long time.

However, contrary to our prediction, prosocial motivation is not associated with entrepreneurial work burnout, and work burnout does not mediate the relationship between prosocial motivation and exit intention. In fact, the relationship between prosocial motivation and work burnout in previous empirical studies is inconsistent, with debating results [164,165,166]. Why this hypothesis is not supported by the results probably is due to the age distribution of the sampled entrepreneurs. Compared with experienced entrepreneurs, young entrepreneurs especially nascent entrepreneurs normally are more excited about starting a new career and managing a business. Prior research suggests a non-linear age trend of entrepreneurial enthusiasm: the entrepreneurial enthusiasm increases with age, peaking at around the age of 35–44 [167]. In this research, over 80% of the respondents are below 45 years old. As a result, their work burnout becomes obscure and difficult to identify.

Furthermore, this research finds that the impact of prosocial motivation on job satisfaction and work anxiety are stronger for male entrepreneurs than female. These results respond to previous research calls for exploring why social ventures are more likely to be started up by men than by women, but the gender gap throughout the entrepreneurial life cycle is not as high as it is in an early stage. Echoing that when gender differences are studied, the results are often shocking and disheartening [168], this study’s findings are remarkable as well, as they unveil the gender stereotype of social entrepreneurs on the one hand, and further imply the complexity of gender equity based on work-related gender stereotypes in the context of social entrepreneurship on the other [169].

Overall, our study provides insights on how prosocial motivations of entrepreneurs influence job satisfaction and work anxiety and consequently, affect exit intention, explicating the complexity in terms of how a social entrepreneur’s typical personality traits can influence her or his intention to exit, while echoing the suggestion that prosocial motivation has limited explanatory power for entrepreneurial exit [27,28,29,30,31]. Based on the findings of this study, the distinctiveness of social entrepreneurs and entrepreneurship is highlighted in a particular way, thus inspiring further studies on the arduousness and complexity. In addition to the contributions to social entrepreneurship studies, this study potentially contributes to the research on entrepreneurial psychology, and furthers our current understanding of the relationship between personality traits and entrepreneurial outcomes [28,30].

### 5.2. Practical Implication

According to our findings, social entrepreneurs tend to experience a lower level of work-related wellbeing, leading to escalation of the exit intention, which in turn, undermines their business sustainability and career development. Thus, social entrepreneurs need to sufficiently be aware of the role of work-related wellbeing, and find proper alternatives to improve it. Furthermore, relevant governmental agencies should provide more support such as relevant policies, facilities, training, and consultation to improve social entrepreneurs’ work-related wellbeing. In addition, based on the findings that male social entrepreneurs may have a lower level of work-related wellbeing compared with female entrepreneurs, relevant supportive policies and actions by governmental agencies become necessary, facilitating social entrepreneurs’ gender equality and better work-related wellbeing and thus the sustainability of social entrepreneurship.

### 5.3. Limitations and Future Research Directions

This research is not without limitations. First, although the relationships among various factors proposed by the theory have been explored in this study, to better understand the sequential influence and causal relations among the variables, longitudinal or experimental design can be employed in future studies. Second, although most of this study’s hypotheses have been supported by the findings, the external validity issue might need to be addressed in further studies, since the data were collected in China which has distinctive features in terms of society, culture, and lifestyle. From another perspective, due to this context’s distinctions, further research may facilitate developing a theoretical model better adaptive to such a context of complexity, thus complementing relevant theories. Third, further studies may need to optimize the data collection approaches such as the self-report questionnaire, as some entrepreneurs (respondents) might deem themselves more optimistic than they truly are, and this changes with age. Fourth, our investigation involved the three dimensions of work-related wellbeing as the mediator, while entrepreneurship, especially social entrepreneurship, requires more input of time and effort, thus allocating more time and space for life to work [14,170], leading to a situation where wellbeing or satisfaction with work and with other life aspects are intertwined [171]. Thus, future studies may need to involve life wellbeing or satisfaction besides work-related wellbeing to enrich our understanding. Finally, although this study supplements the rare quantitative studies in social entrepreneurship research [172,173], a detailed mechanism involving more variables and their relations to uncover how social entrepreneurs’ exit intention is fostered is still unknown, which in turn, may need qualitative approaches to further explore.

## 6. Conclusions

Prosocial motivation and work-related wellbeing play an important role in entrepreneurial exit intention. In this research we have focused on a particular type of entrepreneur—social entrepreneurs, who are aware of the importance of social value creation, embedding relevant activities into their business activities. The model proposed and confirmed by this study indicates that prosocial motivation decreases social entrepreneurs’ work-related wellbeing, which in turn, increases their entrepreneurial exit intention. Moreover, the impact of prosocial motivation on work-related wellbeing largely is stronger for males.

## Figures and Tables

**Table 1:** Sample demographics.

Characteristics	Frequency	Percent (%)
Age		
18–25	19	6.30%
26–35	105	34.90%
36–45	118	39.20%
46–55	59	19.60%
Gender		
Male	145	48.20%
Female	156	51.80%
Marital status		
Married	202	67.10%
Non-married	99	32.90%
Educational Level		
Junior high school	0	0%
High school or equal	3	1%
Junior college	75	24.90%
Bachelor degree	127	42.20%
Postgraduate or above	96	31.90%

**Table 2:** Reliability and AVE of the outer model.

Construct	Items	Factor Loading	α	Rho_A	CR	AVE
Prosocial Motivation (PM)	PM 1	0.865	0.844	0.849	0.895	0.682
	PM 2	0.845	-	-	-	-
	PM 3	0.75	-	-	-	-
	PM 4	0.841	-	-	-	-
Work Burnout (WB)	WB 1	0.585	0.967	0.844	0.949	0.657
	WB 2	0.555	-	-	-	-
	WB 3	0.646	-	-	-	-
	WB 4	0.735	-	-	-	-
	WB 5	0.9	-	-	-	-
	WB 6	0.946	-	-	-	-
	WB 7	0.835	-	-	-	-
	WB 8	0.94	-	-	-	-
	WB 9	0.944	-	-	-	-
	WB 10	0.887	-	-	-	-
Work Anxiety (WA)	WA 1	0.889	0.922	0.951	0.944	0.808
	WA 2	0.926	-	-	-	-
	WA 3	0.904	-	-	-	-
	WA 4	0.875	-	-	-	-
Job Satisfaction (JS)	JS 1	1	1	1	1	1
Exit Intention (EI)	EI 1	0.902	0.93	0.93	0.955	0.877
	EI 2	0.963	-	-	-	-
	EI 3	0.944	-	-	-	-

Note 1: α, Cronbach’s alpha; AVE, average variance extracted; and CR, composite reliability. Note 2: Job satisfaction is a single-item construct.

**Table 3:** Results of discriminant validity by HTMT.

Constructs	EI	JS	PM	WA	WB
EI					
JS	0.499				
PM	0.558	0.348			
WA	0.505	0.101	0.575		
WB	0.053	0.075	0.136	0.079	

Note: PM = prosocial motivation; JS = job satisfaction; WB = work burnout; WA = work anxiety; EI = exit intention.

**Table 4:** Standardized factor loadings and cross-loadings of the outer model.

Items	EI	JS	PM	WA	WB
EI1	0.901	−0.504	0.433	0.389	−0.081
EI2	0.963	−0.470	0.468	0.474	−0.062
EI3	0.944	−0.402	0.481	0.517	−0.049
JS1	−0.489	1	−0.316	−0.105	0.082
PM1	0.437	−0.234	0.867	0.435	−0.283
PM2	0.351	−0.221	0.847	0.472	−0.101
PM3	0.379	−0.292	0.746	0.367	−0.074
PM4	0.456	−0.301	0.842	0.448	−0.185
WA1	0.318	−0.017	0.381	0.89	0.023
WA2	0.409	−0.116	0.467	0.927	−0.048
WA3	0.378	−0.086	0.432	0.904	0.026
WA4	0.588	−0.156	0.552	0.875	0.014
WB1	0.03	0.004	0.01	0.038	0.585
WB10	−0.009	0.059	−0.121	0.075	0.883
WB2	0.042	0.062	0.086	0.107	0.552
WB3	0.086	0.025	0.054	0.148	0.643
WB4	0.016	0.048	−0.048	0.053	0.732
WB5	−0.049	0.018	−0.142	−0.011	0.902
WB6	−0.045	0.075	−0.106	0.038	0.945
WB7	−0.033	0.065	−0.085	0.058	0.833
WB8	−0.049	0.067	−0.161	0.038	0.94
WB9	−0.056	0.053	−0.199	−0.002	0.944

Note 1: PM = prosocial motivation; JS = job satisfaction; WB = work burnout; WA = work anxiety; EI = exit intention. Note 2: The grey cells are the factor loadings of scale items for each construct.

**Table 5:** Summary of inner model results.

Hypotheses	Path Coefficients (β)	*t*-Value	Supported
H1a: PM→JS	−0.319 ***	6.33	Yes
H2a: PM→WA	0.522 ***	8.825	Yes
H3a: PM→WB	−0.200 ^n.s.^	1.318	No

Note 1: PM = prosocial motivation; JS = job satisfaction; WB = work burnout; WA = work anxiety. Note 2: *** *p*-value < 0.001; ^n.s.^ not significant. Note 3: Number of bootstrap samples = 5000.

**Table 6:** Test of mediation effect.

Hypotheses	Path	Direct	Indirect	Total	VAF	Mediation
H1b	PM→JS→EI	0.177 ^n.s.^	0.133 ***	0.31	42.90%	Supported
		(−0.532)	(−4.958)			
H2b	PM→WA→EI	0.177 ^n.s.^	0.183 ***	0.36	50.80%	Supported
		(−0.532)	(−4.133)			
H3b	PM→WB→EI	0.177 ^n.s.^	0.003 ^n.s.^	0.18	16.70%	Not Supported
		(−0.532)	(−0.311)			

Note 1: PM = prosocial motivation; JS = job satisfaction; WB = work burnout; WA = work anxiety; EI = exit intention. Note 2: Number of bootstrap samples = 5000. Note 3: *** *p*-value < 0.001; ^n.s.^ not significant. Note 4: *t*-values are indicated in the brackets. Note 5: VAF = the variance accounted for.

**Table 7:** Multi-group analysis results.

Hypotheses	Path	Pooled N = 301		Group A (Male)		Group B (Female)		Grp A vs. Grp B	Supported
				*N* = 145		*N* = 156			
		β	CI	β	CI	β	CI	*p*-Value	
H4a	PM→JS	−0.334	(−0.421, −0.236)	−0.177	(−0.330, −0.034)	−0.391	(−0.518, −0.251)	0.046	Yes
H4b	PM→WA	0.522	(0.407, 0.626)	0.624	(0.481, 0.729)	0.395	(0.182, 0.551)	0.044	Yes
H4c	PM→WB	−0.200	(−0.316, 0.331)	−0.231	(−0.341, 0.390)	−0.247	(−0.465, −0.140)	0.87	No

Note1: PM = prosocial motivation; JS = job satisfaction; WB = work burnout; WA = work anxiety; EI = exit intention. Note2: β = path coefficient; CI = 95% confidence interval.

## Data Availability

The data will be made available on request from the first author.

## Appendix A

**Table A1:** The Questionnaire.

Construct	Items	Variables	References
Prosocial Motivation (PM)	(1) I care about benefiting others through my work	PM1-PM4	[54]
	(2) I want to have positive impact on others		
	(3) Because I want to have positive impact on others		
	(4) It is important to me to do good for others through my work		
Job Satisfaction (JS)	(1) Generally speaking, I am satisfied with my job	JS1	[140]
Work Anxiety (WA)	(1) I have felt fidgety or nervous as a result of my job	WA1-WA4	[145]
	(2) My job gets to me more than it should		
	(3) There are lots of times when my job drives me right up the wall		
	(4) Sometimes when I think about my job, I get a tight feeling in my chest		
Work Burnout (WB)	When you think about your work overall, how often do you feel the following?	WB1-WB10	[146]
	(1) Tired		
	(2) Disappointed with people		
	(3) Hopeless		
	(4) Trapped		
	(5) Helpless		
	(6) Depressed		
	(7) Physically weak/Sickly		
	(8) Worthless/Like a failure		
	(9) Difficulties sleeping		
	(10) “I’ve had it”		
Exit Intention (EI)	Participants rated the extent to which they would, in the next year?	EI1-EI3	[49]
	(1) Avoid entrepreneurial positions		
	(2) Feel anxious about entrepreneurial positions		
	(3) Feel less excited about entrepreneurial positions		
